# Indications

**Published:** 1880-10

**Authors:** 


### INDICATIONS.

When the captain of a ship at sea notes that the mercury in his barometer is falling, falling, he knows that it does so because there is no longer sufficient atmospheric pressure to keep it up, and he knows also that this state of affairs indicates the near approach of a storm, and, if he is a prudent man, he takes in sail, makes all taut and gets ready to run before the wind, if need be. If he is a fool, he may say to himself, "Oh, I have the best vessel the world ever saw; she has weathered storms before now, and I guess she'll go through this all right." What the net results of this latter plan will be when the storm comes, needs no prophet to foresee.

To-day, we who are passengers, officers and crew upon the great ship of state may well feel filled with alarm at the indications for the future. The political barometer indicates an area of low moral pressure all over the land, and it needs but little skill to read in all our political atmosphere the portends of the coming storm. No use for us to say, "Oh, we have the best government under the sun; we have weathered big storms before now and we'll pull through somehow." To drop metaphor, it must be evident to the most superficial observer that there is a crisis at hand which will sorely try the stability of our institutions and which can hardly pass without leaving some marks of change upon the structure and organization of our government. Happy will it be if these changes shall be affected without revolution and bloodshed.

The cause of this condition of affairs is not far to seek. It is the natural result of our governmental form, methods and traditions. When in the early days of this republic, Jeffersonian ideas gained the ascendency over those of Washington and Hamilton and such clap-trap maxims as "Vox populi vox dei," "All men are born free and equal," and "That government is best which governs least," became seriously engrafted upon the policy of the government, they were popularized and the central authority weakened and the first steps taken in a path which has led slowly but surely downwards towards revolution and anarchy. The voice of the people may be in some slight sense the voice of God when the "people" are all God-fearing, industrious and intelligent.

If all men, *were*, indeed, born free and *equal*, then the people might remain God-fearing, industrious and intelligent and the "government which governs the least" might be "the best" *for such a people.* But the sturdy, intelligent God-fearing men who were "the people" of this country when our government was adopted, are gone, and popular suffrage to-day, so far from being an expression of the voice of God, or even of God fearing men, is through the mistaken liberality of our naturalization laws, too often the voice of ignorance and superstition.

Nor is this the worst. Not only do we welcome the ignorant foreigner to our shores and ask him to take part in making our laws long before he is competent to do so, but we admit the pauper and criminal classes to a share in the government. How often has the vote of the pauper and criminal wards of New York City negatived that of the intelligent taxpayers of the whole state outside of Manhattan Island!

For a long time, [until the Republicans controlled the Freedmen], the Democratic party had almost a monopoly of this class of voters and so long as they were under the control of the old-time leaders of the Democracy, men of education, ability and refinement, who made the platforms and nominations and dictated the policy of the party and then marshalled the masses of the party at the polls as skillful herders run in their cattle to the corrall—the damage was comparatively slight, but when the masses began to be restive under the guidance of the "silk stockings," and to select leaders from among themselves, the danger became more apparent.

A democracy has been aptly described as a government of the most ignorant, because there happens to be most of them;" and there must always be a few of higher intellegence than the many. If we could exclude from the polls, the ignorant, the vicious and the pauper classes, we might hope for better laws and better officers. If the principles of "no taxation without representation" be correct, then, the obverse, "No representation without taxation is equally so." If tax-payers alone had the disposal of the public money, it would be soon economically handled. If the intelligent classes alone had the selection of officers, the selections would be made intelligently.

There is trouble ahead, and there is no way, that we can see, to avoid it but to restrict the suffrage and centralize the powers of government. How can we do this? Not peacefully we fear, and yet it must be done in the near future, and if not peacefully, then it will come through revolution, anarchy and blood.

